# Chameleon sequences—Structural effects

**DOI:** 10.1371/journal.pone.0315901

**Published:** 2025-04-22

**Authors:** Mateusz Slupina, Katarzyna Stapor, Leszek Konieczny, Krzysztof Gądek, Piotr Nowakowski, Irena Roterman

**Affiliations:** 1 ALSTOM ZWUS Sp. z o.o. Modelarska, Katowice, Poland; 2 Faculty of Automatic, Electronics and Computer Science, Department of Applied Informatics, Silesian University of Technology, Gliwice, Poland; 3 Chair of Medical Biochemistry, Jagiellonian University - Medical College, Krakow, Poland; 4 Sano Centre for Computational Medicine, Kraków, Poland; 5 Department of Bioinformatics and Telemedicine, Jagiellonian University -Medical College, Krakow, Poland; Thapar Institute of Engineering and Technology: Thapar Institute of Engineering and Technology (Deemed to be University), INDIA

## Abstract

The predisposition of amino acids towards accepting the appropriate secondary structure form is ambiguous. The identified sequences (6–12 aa in length – ChSeq data base) of the chameleon type (the same sequence accepting different secondary structures) constitute a puzzle that makes it difficult to indicate the initial conformation in a chain with a given amino acid sequence. The analysis of proteins presented in this paper uses the hydrophobicity distribution in protein body as the criterion for comaparable analysis of the status of helica/Beta-structural chameleon fragments in pairs of proteins. The sub-base is the object of analysis containg the proteins representing the organisation of hydrophobicity in one protein of the pair as ordered according to micelle-like organisation (hydrophobic core with polar surface) and the second one in pair with disordered hydrophobicity organisation. The status of chameleon sections appears to represent local organisation of hydrophobicity highly accordant in both proteins in chameleon pair independently on the status of the structural unit they belong to. The fuzzy oil drop model (FOD) in its modified form (FOD-M) is applied for analysis. This work aims to verify the hypothesis assuming the subordination of the form of secondary structure to the superior goal of obtaining a hydrophobicity distribution suitable for the given biological activity of the protein, ensuring biological functionality. Secondary structure is not an aim by itself. It is shown, that the main goal is to reach the structure representing specific activity. Secondary structure is a means to achieve this goal.

## Introduction

Predicting protein structure from a specific amino acid sequence has been a challenge for many years [1]. Progress in this field is monitored as part of the CASP (Critical Assessment of proteins Structure Prediction) project [2,3]. Recently, significant progress has been recorded with the introduction of methods based on Artificial Intelligence (AI) [4]. The deep learning technique applied in AlphaFold model allows the prediction the structure of any protein for given sequence. The method delivering the correct structure even for proteins with no known similar structure is available. It was proven by the CASP14 [4] where this method delivered best models with large distance in respect to other paricipants. The goal in form of receiving the correct structure for given amino acids sequence appears reached. However the question concerning the mechanism of folding is still without the solution. Our model presented below is aimed on the search for the answer to the question: Why the proteins fold the way they do? [5].

In the early stages of the project, the prediction of sections adopting a certain secondary structure was also a challenge. This discipline was abandoned as early as at the CASP6 stage [6]. This took place as a result of achieving increasingly better results for tertiary structure prediction and because of the identification of chameleon sequences with identical amino acid sequence (sections of 7–12 aa) representing opposite structures: helical and β-structure [7].

A database named ChSeq has been created that collects pairs of proteins with chameleon sequences [8]. The access to this large data base enables their accurate analysis. In this regard, it is also important to assess the predisposition of the corresponding tetrapeptide sequences to generate a specific secondary structure [9,10].

In the present study, 298 proteins from said database were analysed using the distribution of hydrophobicity within a given structural unit (chain, domain) as a criterion for assessing the degree of structural similarity of units (polypeptide chain, domain) containing chameleon sections. The status of structural units in protein pairs and the status of chameleon sections are assessed. The assessment of the status of a structural unit allows differentiating hydrophobicity distribution within that unit. The chameleon section status, on the other hand, expresses the contribution of the hydrophobicity distribution of a given chameleon section to achieving the distribution at the level of the entire unit. The status of a section is sometimes consistent with the status of the entire unit, which is interpreted as a contribution to the construction of the arrangement within the unit. The status of the section distinct from that of the structural unit suggests a specific role for the section as the carrier of a local disorder of the unit’s arrangement.

In addition, a comparison of the status of a pair of chameleon proteins – it may be uniform for both proteins or different – reveals a variation of the “end products”, which often implies a differentiation of biological function. It is also possible to compare the status of chameleon sections within comparable or different statuses of the unit as a whole.

Such an analysis is made possible by the use of the fuzzy oil drop model (FOD-M) [11,12].

In the present work, the sub-base of ChSeq was analysed. Pairs of proteins representing different statuses were selected: one representing a micelle-like arrangement of hydrophobicity (a centrally located hydrophobic core with a polar surface shell); the other, on the contrary, a distribution different from micelle-like status. The exact criterion is described in the Materials and Methods section.

The hypothesis – supported by the following analysis – indicates a subordinate role of the secondary structure to the superior objective of achieving a function-related distribution of hydrophobicity throughout the structural unit. The appropriate form of secondary structure present in a protein is a means to achieve a given status and not an end in itself.

The model treating the folding process as dependent on internal force field (non-bonding interaction between atoms in the protein) and also dependent on the external force field (influence of environment) directing the folding process toward the specific organisation of hydrophobicity distribution in protein body. The model is aimed to explain for example the specificity of membrane prtotein folding or folding process requiring the presence of prefoldin [13], chaperone [14] and chaperonin [15].

It is shown that the form of secondary structure is adopted to overriding aim which is the specific hydrophobicity distribution in certain part of protein body. This is why the chameleon sequences adopt different forms reaching the similar goal – the hydrophobicity distribution necessary to ensure the expected biological activity.

## Materials and methods

### Description of the FOD-M model used

The analysis starts by identifying the structural unit in which a chameleon section is present. Domain identification was performed based on the CATH classification [16]. These classification is available in in the PDBSum database [17].

The status of the structural unit was assessed using the fuzzy oil drop FOD model in a modified version – FOD-M – that takes into account the contribution of the environment to protein folding [18].

This model assumes that a given protein structure is obtained as a result of the optimisation of internal non-binding interactions (internal force field) and the active participation of the local environment (external force field) directing the structuring process. This active participation of environemnent allows achieving the hydrophobicity distribution within the protein representing the specificity of the immediate vicinity of the folding protein. It is assumed that a polar water environment directs the folding process towards generating a micelle-like arrangement: hydrophobic core and polar surface. A 3D Gaussian function is used to describe such an idealised distribution. The actual distribution within the protein may be different. The degree of dissimilarity can be quantified by comparing the distribution according to the 3D Gaussian function with the distribution resulting from inter-amino acid interactions. A quantitative measure of the degree of similarity determines the status of a given structural unit (complex, individual chain, domain).

The two functions used are

1. Distribution theoretical – Ti – an idealised distribution fulfilling the criteria of a micelle-like system with a centrally located core and a gradually decreasing level of hydrophobicity up to zero at the surface. This distribution is expressed by a 3D Gaussian function spanning the protein body. The value of this function at the position of the effective atom (the averaged position of the atoms comprising a given amino acid) expresses the expected idealised level of hydrophobicity at a given location of the protein.

$$H_{i}^{T}=\frac{1}{H_{sum}^{T}}\exp\left(\frac{-{\left(x_{i}-\bar{x}\right)}^{2}}{2\sigma_{x}^{2}}\right)\exp\left(\frac{-{\left(y_{i}-\bar{y}\right)}^{2}}{2\sigma_{y}^{2}}\right)\exp\left(\frac{-{\left(z_{i}-\bar{z}\right)}^{2}}{2\sigma_{z}^{2}}\right)$$ (1)

The *x*_*i*_*, y*_*i*_*, z*_*i*_ – coordinates defining the position of i-th effective atom (averaged position of atoms belonging to particular amino acid), $\bar{x}$, $\bar{y}$ and $\bar{z}$ express the mean values of x, y and z coordinates respectively, values of the parameters *σ*_*x*_*, σ*_*y*_ and *σ*_*z*_ are adjusted to the size and shape of the protein examined. The $H_{i}^{T}$ value defines the idealised, theoretical level of hydrophobicity at i-th residue, assuming a distribution consistent with a micelle-like system expressed as T_i_ in this paper. The $H_{sum}^{T}$ expresses the sum of all $H_{i}^{T}$ making the $H_{i}^{T}$ values normalised. The formula for Gauss function is commonly known. The first use of the 3D Gauss function in FOD model was reported in [11].

1. The observed distribution – Oi – resulting from hydrophobic inter-amino acid interactions. The function introduced by M. Levitt [19] was used here. This interaction is expressed by the function:

$$H_{i}^{O}=\frac{1}{H_{sum}^{O}}\sum_{j} \{\begin{array}{l} \left(H_{i}^{r}+H_{j}^{r}\right)\left(1-\frac{1}{2}\left(7{\left(\frac{r_{ij}}{c}\right)}^{2}-9{\left(\frac{r_{ij}}{c}\right)}^{4}+5{\left(\frac{r_{ij}}{c}\right)}^{6}-{\left(\frac{r_{ij}}{c}\right)}^{8}\right)\right)\;forr_{ij}\leq c\\ 0,\;forr_{ij}>c \end{array}$$ (2)

Where r_ij_ – distance between positions of effective atoms, c – cutoff distance for these interactions taken according to 9Ǻ [19]. The magnitude of interaction depends on the intrinsic hydrophobicity of interacting amino acids – $H_{i}^{r}$ (an arbitrary scale can be used) [20].

The $H_{\begin{array}{l} i \end{array}}^{O}$ values determine the level of hydrophobicity present at the position of i-th amino acid (called *O*_*i*_ later on in this paper). The $H_{sum}^{O}$ expresses the sum of all O_i_ making them normalised. This is the observed level corresponding to the actual status of the amino acid in question, taking into account the immediate environment of the given amino acid.

The comparison of these two distributions (after prior normalisation – first positions in eqs 1 and 2) allows a quantitative assessment of the degree of similarity of the *O* (observed) distribution to the *T* (theoretical) distribution. The divergence entropy defined by Kullback-Leibler [21] is used for this purpose.


$$D_{KL}(P|Q)=\sum_{i=1}^{N}P_{i}\log_{2}\frac{P_{i}}{Q_{i}}$$ (3)

Where the *P* distribution – the distribution examined – in the case of the FOD model this is the *O* distribution, the *Q* distribution is the reference distribution, in the FOD model – the T distribution. The determined *D*_*KL*_ value for the relationship (*O | T*) is not interpretable. Therefore, a second reference distribution *R* is introduced with *Ri = 1/N*, where *N* is the number of amino acids in the chain. This is a unified distribution for the whole molecule, where each amino acid is assigned an equal *R*_*i*_ value. This reference distribution represents a structure with no variation in hydrophobicity levels (hydrophobic core absent) in the protein body. The *D*_*KL*_ value for the (*O | R*) relationship determines the degree of closeness of the *O* distribution to a distribution devoid of the presence of a hydrophobic core (uniform distribution throughout the protein).

The *D*_*KL*_*(O | T)* <  *D*_*KL*_*(O | R)* relationship is interpreted as stating the presence of a hydrophobic core. The degree of fit of the *O* distribution to the *T* distribution is expressed by the *RD* (Relative Distance) parameter determined according to the equation:


$$RD=\frac{D_{KL}\left(O\text{|}T\right)}{D_{KL}\left(O\text{|}T\right)+D_{KL}\left(O\text{|}R\right)}$$ (4)

Eq. 4 has been introduced by Authors of the FOD-M model [22].

### A value of *RD* <  0.5 is interpreted as the presence of a hydrophobic core.

The identification of proteins with a status very close to the *T* distribution confirms the correctness of the model used. These are proteins belonging to the group: downhill, fast-folding, ultra-fast-folding and antifreeze type II proteins [23].

**Fig 1:**
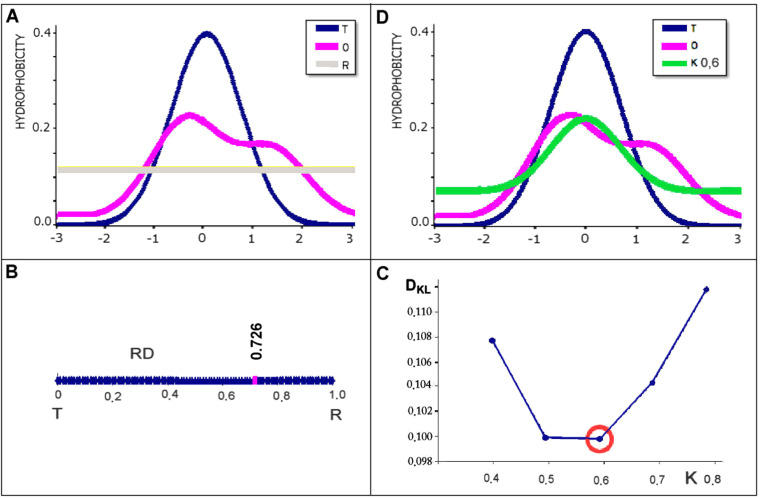
Visualisation of the model used. A – example of *T, O* and *R* distribution – form reduced to 1D. B – determined value of parameter *RD* =  0.725, suggesting micelle-like status. C – method of determining the value of the *K* parameter – the value corresponds to the minimum *D*_*KL*_ value for the (*O | M*) relationship. D – summary of *T, O* and *M* distributions for the *K* value determined as per C.

A structural unit can be defined respective to the problem under analysis: multi-chain complex, single chain or domain. A 3D Gaussian function is generated for each listed structural unit. It is also possible to determine the status of a selected chain section and its importance (role) in the construction of a micelle-like distribution within a given structural unit. A value of *RD* <  0.5 for the selected fragment (this can be the status of a single chain in a complex or a chain fragment against a randomly selected structural unit) indicates the participation of this fragment in the construction of a generally unit type of hydrophobicity distribution. A value of *RD* >  0.5 for a selected chain fragment indicates its local inadequacy in the construction of the hydrophobic core common for the entire unit. When analysing the status of a selected structural unit fragment, the set of *O*_*i*_*, T*_*i*_ and *R*_*i*_ values is subjected to a normalisation procedure. This part of the model is visualised in Fig 1.A, where the *O* distribution (pink) is compared with the reference *T* distribution (blue – hydrophobic core present) and with the reference *R* distribution (gray – uniform distribution) (Fig 1.A). The determined value *RD* = 0.725 for the given example is presented on the axis of variation of *RD* values (Fig 1.B). It suggests treating the *O* distribution as not meeting the micelle-like criterion.

The status of the O distribution, expressed by the *RD* parameter, may also be computed for a fragment of the given structural unit (chain in complex or domain in chain). Such *RD* values can be calculated using eq. 3 and eq. 4 following normalizastion of *O*, *T* and *R* distributions for the selected fragment. Under these assumptions, *RD(FR)* (where *FR* stands for “fragment”) determines the influence of the given fragment upon the conformation of its parent structural unit, and its contribution to that unit’s overall distribution of hydrophobicity. In this work chain fragments are studied in the context of chameleon sequences. From the *T*, *O* and *R* profiles calculated for the complete structural units we select fragments of interest, then renormalize their corresponding distributions, obtaining *T*_*i*_, *O*_*i*_ and *R*_*i*_ – under the assumption that the sum of all *T*_*i*_ values for the given fragment should be equal to 1, and likewise for *O*_*i*_ and *R*_*i*_. These distributions serve as the basis for computing *RD(FR)* (see Supplementary Materials for further examples).

The model discussed herein has been used to assess the status of a structural unit where a chameleon fragment is present and the status of the section itself with identical sequence in two different proteins. The structural units used in the present analysis follow the CATH classification [16]. The status of chameleon sections is determined relative to the structural unit in which the section is present.

The modified FOD-M model takes into account the presence of a polar water environment modified by other compounds (hydrophobic ones in particular) for a given protein, which is assumed to influence the folding process of the protein not necessarily directing the structuring towards the generation of a centric hydrophobic core. The degree of mismatch in the *O* distribution against the *T* distribution may be due to different characteristics of the environment. The construction of a centric core with a polar shell is characteristic of an aqueous environment interacting with bi-polar molecules, which is what all amino acids are (with a variable ratio of polar to hydrophobic parts).

The presence and influence of the external field on the formation of the hydrophobicity distribution within the protein molecule was defined, starting from the characteristics of membrane proteins, where the hydrophobic part of the membrane expects the exposure of hydrophobic amino acids on the protein surface. In addition – especially for membrane proteins acting as a channel – a concentration of polar residues in the central part of the protein is expected. Therefore, the description of the force field in a membrane protein is expressed by a function complementary to the Gaussian function:


$$M_{i}=T_{max}-T_{i}$$ (5)

Eq. 5. has been introduced by Authors of FOD-M model [22].

In fact, the analysis of membrane proteins suggests the generic use of the following equation to record the force field based on hydrophobicity:


$$M_{i}=T_{i}+{\left[K\;*{\left(T_{max}-T_{i}\right)}_{n}\right]}_{n}$$ (6)

Eq. 6. has been introduced and applied by Authors of the FOD-M model [22].

In this field record, there is a water-derived field (3D Gauss function – *T*_*i*_) modified by the presence of a complement field (*T*_*MAX*_*-T*_*i*_) to a degree expressed by the value of the parameter *K.* The parameter *K* can take the value K = 0.0 for proteins with a hydrophobicity structure compatible with a centric core and a polar surface [23] up to a value *K* > 3 for proteins showing structuring far from the micelle-like form [18].

The value of the *K* parameter is determined as part of the procedure to find the minimum value of *D*_*KL*_*(O | M)* (Fig 1.C), assuming that the value of the *K* parameter thus determined defines the *M* distribution most similar to the *O* distribution. Thus, it can also indicate the degree of dissimilarity (with respect to the polar water field) of the external field for protein folding (Fig 1.D).

The interpretation of the FOD-M model parameters is as follows:

1. The *RD* value indicates the extent to which the micelle-like system has been reproduced in the protein structure. High *RD* values indicate a significant dissimilarity of the distribution to the micelle-like distribution with a centric hydrophobic core. It also means an approximation of the *O* distribution to the uniform *R* distribution, lacking the central nature of hydrophobicity in the protein.
2. The *K* value determines the degree to which factors alter the specificity of the aqueous environment by introducing a hydrophobic factor in particular. The higher the *K* value, the weaker the influence of the polar water environment on the structuring directed towards the generation of the hydrophobic core.

### Analysis of the status of chameleon chain fragments

All proteins analysed here are characterised according to the following procedure:

1. A structural unit is identified where a chameleon fragment is present (according to the CATH classification 16]) available in the PDBSum database [17].
2. For the identified structural unit (chain or domain), the *RD* value is determined to obtain the degree of micelle-like system reconstruction.
3. For this unit, the *K* value is also determined to identify the degree of dissimilarity of the environment affecting the structuring in relation to an external force field such as polar water [5,24].
4. For the chameleon section, the *RD* value is determined to identify the form of the local contribution of the given fragment in relation to the hydrophobicity distribution within the structural unit. Before the *RD* value is determined, the *T* and *O* and *R* profile sections (for chameleon fragments) shall be normalised.

**Fig 2:**
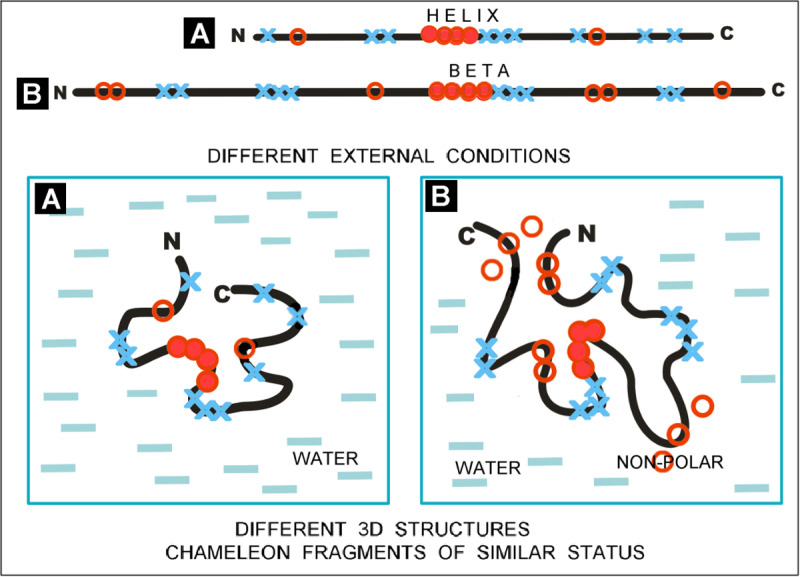
The summary of the influence of the specific environment on the structuring of a polypeptide chain with a hydrophobicity (red O)/polarity (blue X) distribution of amino acids – aa sequences with varied hydrophobicity – top line with a highlighted different secondary structure for chameleon sections (boxes). A – the effect of environment with varying polarity on the structuring of the sequence listed as 1. B – the effect of environment with varying polarity on the structuring of the sequence listed as 2. HHH… denotes a helical fragment; BBB… denotes a beta-structural fragment.

To visualise the model used, an abstract example of the influence of the environment on the formation of the structure with varied secondary structure for a chameleon section is used (Fig 2).

Fig 2 is intended to justify the needed variation in the effects of polypeptide chain folding depending on the environment. The identification of the different status of the structural units (expressed by the *RD* and *K* parameters) – as assumed in the FOD-M model – is precisely due to the influence of the environment and not limited to the characteristics of the amino acids themselves included in the compared sections with identical sequence.

In both of the examples presented, the essence lies in adjusting the status of the polypeptide chain section in relation to the specificity of the local environment during folding. Obtaining a status within the hydrophobic field that is compatible with it can be guaranteed by an appropriate form of secondary structure. Secondary structure is thus a means to an end and not an end in itself.

### Data used for analysis

The complete database of proteins with 7 aa chameleon fragments under the stringent criterion numbers 777 items. Analysing such a large dataset in a single study would cause confusion – hence the ChSeq database has been divided into three portions:

A – both structural units sharing the given chameleon fragments satisfy RD <  0.5, which means that their conformation is micelle-like (in preparation).
B – both structural units sharing the given chameleon fragments satisfy RD >  0.5, which means that their conformation diverges from a micelle [25].
C – one structural unit satisfies RD >  0.5 while for the other unit RD <  0.5, which means that the conformational properties of both structures vary.

The presented analysis focuses on the dataset defined in item C above – which consists of 232 ChSeq structures.

The ChSeq database available at [8] was used as the source for pairs of proteins with chameleon sequences representing different: helical and β-structural forms for chameleon sequence fragments (6–12 aa). The structure of the proteins listed in the ChSeq database was taken from the PDB database [26].

**Fig 3:**
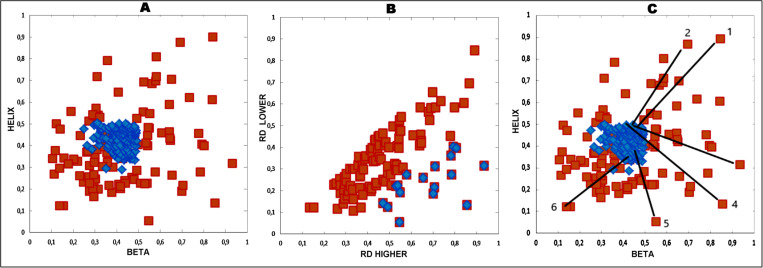
The characteristics of the sub-base of ChSeq base restricted to proteins showing a varied status: one protein in a pair with *RD* >  0.5; the other protein in a pair with *RD* <  0.5. A – scatter diagram for the system: X-axis – *RD* values higher in the pair, Y-axis – *RD* values lower in the analysed pair. Red points – the status of structural units containing chameleon sections, blue points – the status of chameleon sections (RD(FR)). B – the identification of outliers (red points) – representatives with the highest degree of divergence from the equal status are discussed in detail later in the paper. For each point, the status is expressed by RD(FR). C – the identification of pairs of proteins represented in the detailed analysis. Here, the points with the highest divergence from a linear relationship and dots representing pairs with extreme statuses for maximal *RD* (examples 1,2) and minimal *RD* (example 7) were selected. Red dots – as in A, blue dots – as in A. Green lines link the status of of chameleon fragments (blue dots – RD(FR)) with the status of structural units (red dots - RD).

The pairs of chameleon proteins provided in Table 1 are those that represent the extremal relations of RD values describing the status of chameleon fragments (Fig 3.C). The list includes the pairs which extremely differ in respect to assumed hypothesis of comparable status of both structural forms (points: 3–6). This list includes also the examples of extremal status expressed by RD which comparable in both chameleon fragments (points: 1,2 and 7). The points 1 and 2 on Fig 3.C represent the pairs in which both chameleon fragments represent very high RD values (the status far in respect to micelle-like organisation of hydrophobicity in discussed fragments). The point 7 represents the pair of proteins with both chameleon fragments highly accordant with micelle-like local organisation. In the Results section, the criterion for selecting the proteins presented in Table 1 will be explained in detail (Fig 3.C).

**Table 1:** The characteristics of pairs of proteins discussed in detail in the present work. The criterion for the selection of the proteins listed is the relationship of the status of the two proteins towards the system expressing an equal status for both proteins in the pair (outlier points – explained in the Results section).

BETA			SEQUENCE	HELIX		
PDB ID	FUNCTION	Source org.		Source org.	FUNCTION	PDB ID
3N2S [27]	Oxidoreductase	Bacteria	LFGLAVG	Human	Transferase	2Q80 [34]
2YGL [28]	Hydrolase	Bacteria	LVLGGAL	Bacteria	Transferase	2PZI [35]
2J6R-B [29]	Cell adhesion	Ecoli	SAAYALG	Bacteria	DNA binding	4JW3-C [36]
2P8U [30]	Transferase	Human	GAVALLI	Fruit fly	Hydrolase	3LXU-X [37]
3U5E [31]	Ribosome	Backers	GLRSLTT	Human	Cytokine	1EER [38]
1WXX [32]	Transferase	Bacteria	GGILATA	Ecoli	Lyase	1SZQ [39]
4C5I [33]	Transcription	Human	VATVTRI	Mouse	Hydrolase	3UNF-H [40]

### Programs used

VMD programm was used for 3D presentations of proteins structures [41,42].

The program allowing calculation of *RD* is accessible on GitHub platform: https://github.com/KatarzynaStapor/FODmodel and on the platform https://hphob.sano.science. A detailed description of the procedure for computing *RD* (for the entire structural unit as well as for chameleon fragments) is provided as part of Supplementary Materials.

## Results

### Characteristics of the selected sub-set

The number of pairs of proteins meeting the given criterion (RD for one protein in the pair <  0.5; for the other – *RD* >  0.5) is 298 (Fig 3.A – red points). The relationship of the status of chameleon sections is expressed by a correlation coefficient of 0.534 for the ordered system (on the X axis, the *RD* values are higher, on the Y axis, the *RD* values are lower – Fig 3.A – blue points). After eliminating outliers (Fig 3.B), the correlation coefficient =  0.707. It should be noted that the number of outlier points is 28, which represents 10% of the total sub-base analysed. Outlier points were eliminated until a correlation coefficient of >  0.7 was reached.

The value of the correlation coefficient measures the degree of similarity of the hydrophobicity distribution in the chameleon sections regardless of the status of the structural unit to which they belong.

The high value of the correlation coefficient with relatively few outlier points suggests the similarity of the hydrophobicity ordering of chameleon fragments (different secondary structures) despite the significantly different status of the structural units to which they belong.

Pairs whose status stands out in comparison to the others are present in the analysed sub-base. These distinguished pairs are identified by the arrangement of points on the scatter diagram (Fig 3.C). The pairs of proteins (structural units) subjected to detailed analysis are indicated. This group (Table 2) contains examples with a very high consistency at a very high *RD* value (examples 1 and 2) and an example with a very high consistency of the status in a pair of chameleon sections with a high consistency with a micelle-like distribution (example 7 – Fig 3.C, Table 2). A set of pairs of proteins with chameleon sections representing different statuses within their structural units were also analysed in detail (examples 3–6, Fig 3.C and Table 2).

**Table 2:** The characteristics of selected examples of proteins showing extreme positions towards the equal status relationships of chameleon sections within structural units. The *RD* and *K* values for structural units and the *RD(FR)* values for determining the status of chameleon sections within a structural unit are given. The first column contains the identifier used in Fig 3.C.

BETA				SEQUENCE	HELIX		
NR	PDB-ID	Domain – RD/K	RD(FR)	SEQUENCE	RD(FR)	Domain – RD/K	PDB - ID
1.	3N2S	0.432/0.28	0.908	LFGLAVG	0.895	0.640/0.93	2Q80
2.	2YGL	0.435/0.32	0.883	LVLGGAL	0.867	0.504/0.41	2PZI
3.	2J6R-B	0.627/0.73	0.938	SAAYALG	0.264	0.354/0.11	4JW3-C
4.	2P8U	0.564/0.57	0.931	GAVALLI	0.247	0.494/0.40	3LXU-X
5.	3U5E	0.468/0.31	0.911	GLRSLTT	0.241	0.548/0.45	1EER
6.	1WXX	0.476/0.32	0.866	GGILATA	0.190	0.602/0.58	1SZQ
7.	4C5I	0.462/0.28	0.167	VATVTRI	0.152	0.540/0.33	3UNF-H

### Analysis of selected examples

#### Status of chameleon sections comparable – high RD values.

**Fig 4:**
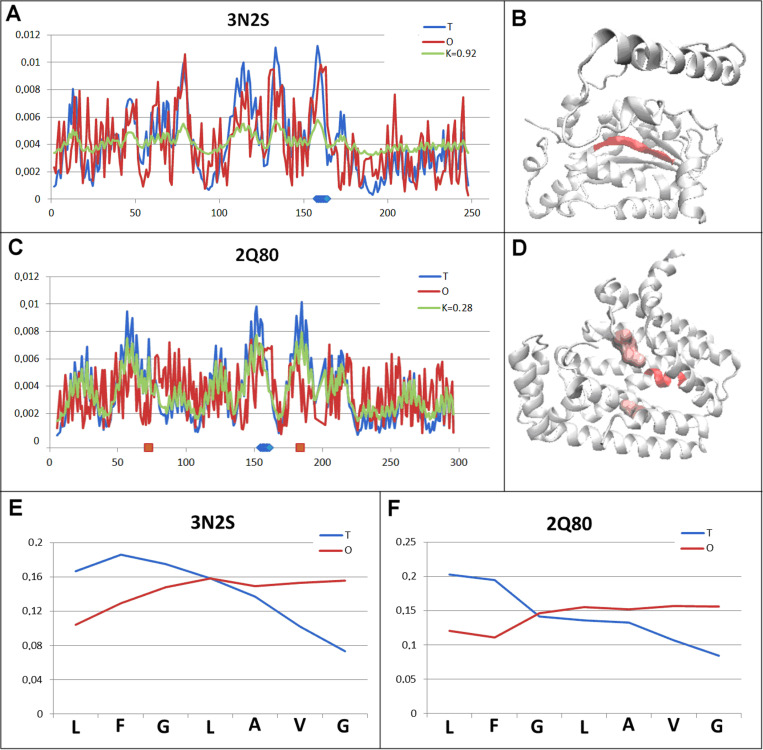
Pair characterisation (example 1 in Fig 3.C) – the pair discussed is on the position 617 in ChSeq data base. A – *T, O* and *M* distribution for the *K* value (given in the legend) describing the status of oxidoreductase (3N2S). B – 3D representation of the structural unit for which the parameters have been determined with the chameleon section (red) highlighted – 3N2S, C – *T, O* and *M* distribution for the *K* value (given in the legend) describing the status of transferase (2Q80). D – 3D representation of the structural unit for which the parameters have been determined with the chameleon section (red) highlighted – 2Q80. Additionally, the positions of the catalytic residues are highlighted in pink. E – T and O distribution for the chameleon section in oxidoreductase (3N2S). F – set of T and O distributions for the chameleon section in transferase (2Q80). The blue dots on X-axis in A and C – chameleon fragment.

The examples represented as examples 1 and 2 in Fig 3.C are proteins with a comparable status of structural units barely exceeding the micelle-like status. A slight overrun of the discriminating threshold (RD = 0.5) suggests the presence of a hydrophobic core and a polar surface (Fig 4.A and 4.B). The chameleon sections in this pair of proteins show a status with very high, nearly equal values of *RD* parameters expressing a very high mismatch between the local distribution and the micelle-like arrangement (Fig 4.C and 4.D). This mismatch is expressed by the opposite of the expected distribution (Fig 4.E and Fig 4.F). This occurs regardless of the secondary form represented by a given chameleon section. Both proteins are enzymes, where the record of encoded biological activity is expected in the specific structuring of the protein. In both examples discussed here, the chameleon section located at the central part of the protein, showing an extremely high local mismatch to the micelle-like arrangement with the status of the whole structural unit close to this state suggests a significant contribution of this chameleon section to the introduction of the specific instability of the whole enzyme molecule required for the catalytic process (Fig 4.B and 4.D). In the case of oxidoreductase (3N2S), the catalytic residues are not indicated, whereas in the case of transferase (2Q80), the location of the catalytic residues allows the identification of the chameleon section as representing the structuring of the bottom of the substrate-binding cavity (Fig 4.D). The positioning of the chameleon section in such a specific location towards the catalytic reaction cavity probably has a specific task.

**Fig 5:**
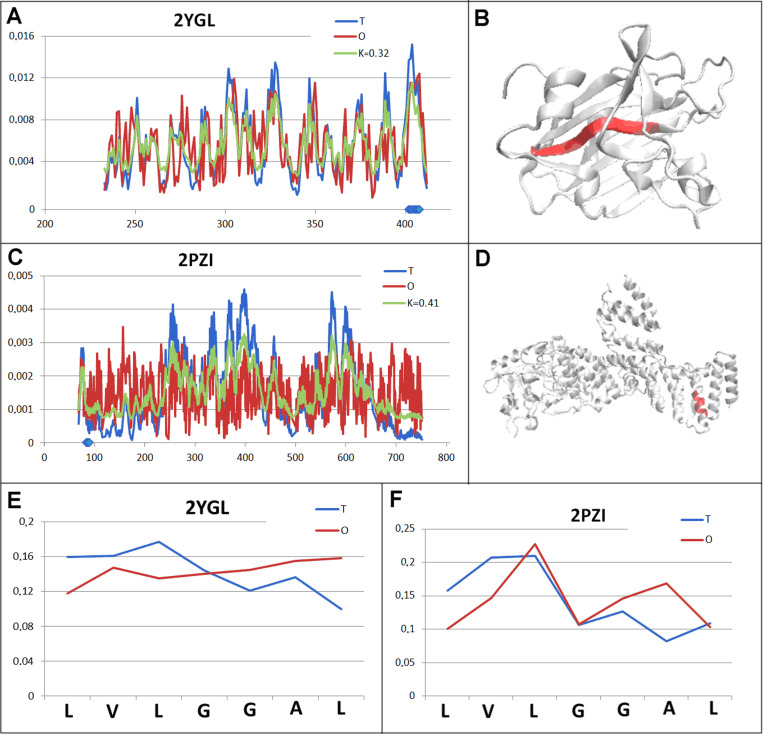
Pair characterisation (2 in Fig 3.C) - – the pair discussed is on the position 243 in ChSeq data base. A – *T, O* and *M* distribution for the *K* value (given in the legend) describing the status of hydrolase (2YGL). B – 3D representation of the structural unit for which the parameters have been determined with the chameleon section (red) highlighted – (2YGL). C – *T, O* and *M* distribution for the *K* value (given in the legend) describing the status of transferase (2PZI). D – 3D representation of the structural unit for which the parameters have been determined with the chameleon section (red) highlighted – 2PZI. E – T and O distribution for the chameleon section in hydrolase (2YGL). F – set of T and O distributions for the chameleon section in transferase (2PZI) The blue dots on X-axis in A and C – chameleon fragment.

The second set representing a system very similar to that discussed above is the hydrolase (2YGL) and transferase (2PZI) set. This pair, similarly to the previous one, represents a status very close to the system with *RD* =  0.5 (Fig 5.A and 5.C). In the case of hydrolase, the chameleon section occupies a central position (Fig 5.B), probably introducing the potential for local instability (the micelle-like system is assumed to introduce stability based on the presence of a hydrophobic core). The chameleon section shows a different positioning, occupying a surface location in a highly extended structural unit (Fig 5.D).

Similar to the previous pair of chameleon proteins, the chameleon section status analysis shows the O distribution exhibiting an opposite arrangement to that of the expected T distribution (Fig 5.E and 5.F).

#### Examples of chameleon sections with an extremely different status (expressed by the *RD* value).

This group of three examples includes a pair: representing cell adhesion (2J6R) and DNA binding (4JW3). The differentiating feature of the proteins in question is the type of target molecule in the complexation process.

**Fig 6:**
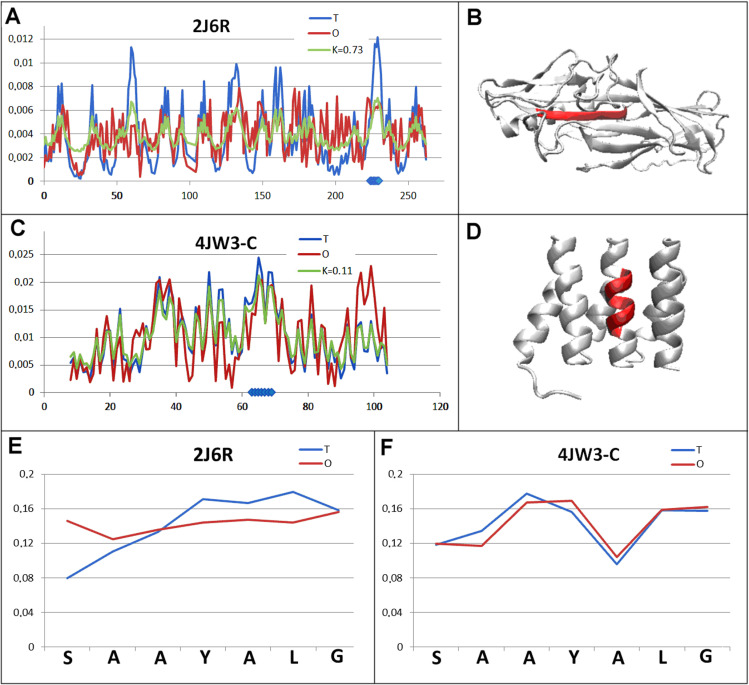
Pair characterisation (3 in Fig 3.C) - – the pair discussed is on the position 458 in ChSeq data base. A – *T, O* and *M* distribution for the *K* value (given in the legend) describing the status of cell adhesion (2J6R). B – 3D representation of the structural unit for which the parameters have been determined with the chameleon section (red) highlighted – (2J6R). C – *T, O* and *M* distribution for the *K* value (given in the legend) describing the status of DNA-binding protein (4JW3). D – 3D representation of the structural unit for which the parameters have been determined with the chameleon section (red) highlighted – 4JW3. E – T and O distribution for the chameleon section in cell adhesion (2J6R). F – set of T and O distributions for the chameleon section in DNA-binding protein (4JW3). The blue dots on X-axis in A and C – chameleon fragment.

The cell adhesion protein shows a hydrophobicity distribution significantly different from the micelle-like arrangement, where the chameleon section introduces a significant (extremely high) degree of order dissimilarity in relation to the micelle-like arrangement. The residue of 225A (included in the chameleon section) is indicated as interacting with the second chain within the dimer (strand-swapped complexation [29]) (Fig 6.A). Complexation of this type requires flexibility of the sections building the interface. The chameleon section represents the status with the highest *RD* value within the group of proteins in question (Table 2), as confirmed by the summary of the *T* and *O* distributions (Fig 6.E).

The second protein of this pair is a DNA-binding protein with a micelle-like arrangement within the structural unit (Fig 6.C). Similarly, the status of the chameleon section shows a very high local match with the arrangement represented by the T distribution (Fig 6.F).

The specificity of the chameleon section within the DNA-binding protein (4JW3) is the involvement of this section in the interaction with the chains that form the quaternary structure consisting of four chains [36]. If it were assumed that the inter-chain interaction takes place via hydrophobic interactions, then the exposure of hydrophobic residues would represent a preparation for such an interaction. The residues S63, S64, Y67 and the neighbouring F60 suggest the involvement of hydrogen and hydrophobic bonds in the generation of the interface (Fig 6.E). The T and O distribution characteristic of the amphipathic helix is seen in Fig 6. F, exactly matching expectations from the point of view of matching hydrophobicity levels – surface-wise and directed to the inside of the molecule. There is no information on the involvement of the section in question in the interaction with DNA.

**Fig 7:**
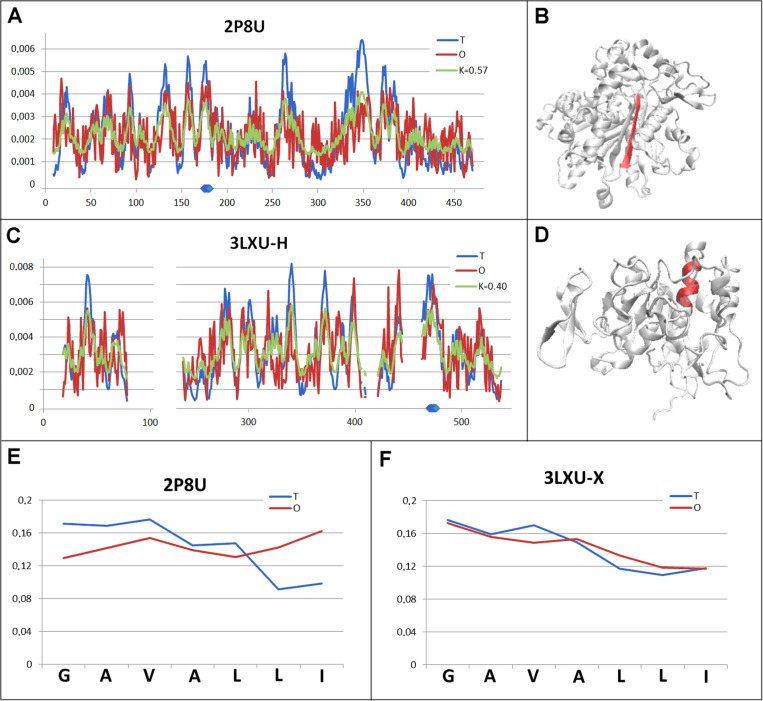
Pair characterisation (4 in Fig 3.C) - – the pair discussed is on the position 583 in ChSeq data base. A – T, O and M distribution for the K value (given in the legend), describing the status of transferase (2P8U). B – 3D representation of the structural unit for which the parameters have been determined with the chameleon section (red) highlighted – (2P8U). C – T, O and M distribution for the K. value (given in the legend), describing the status of hydrolase (3LXU). D – 3D representation of the structural unit for which the parameters have been determined with the chameleon section (red) highlighted – 3LXU. E – T and O distribution for the chameleon section in transferase (2P8U). F –T and O distributions for the chameleon section in hydrolase (3LXU). The blue dots on X-axis in A and C – chameleon fragment.

Another set of chameleon proteins representing a similar arrangement to the previous one is a pair of proteins: transferase (2P8U) and hydrolase (3LXU). The status of both proteins – structural units varies (*RD* > 0.5 for transferase and *RD* < 0.5 for hydrolase) (Fig 7.A and 7.C). The distribution of hydrophobicity levels in transferase shows a cavity typical of enzymes (positions around 300 and also position 260), expressed as a local hydrophobicity deficit. The chameleon section in question also shows a local hydrophobicity deficit (Fig 7.A). The *RD* value for this chameleon section is extremely high (Fig 7.E), showing opposing levels of hydrophobicity at all positions of this section.

A completely different status is represented by both the structural unit and, above all, the chameleon section itself, showing a very high match between the O distribution and the T distribution (Table 2, Fig 7.F). Locally, this match is very high, yet it takes place right next to a section showing a local deficit (Fig 7.C).

**Fig 8:**
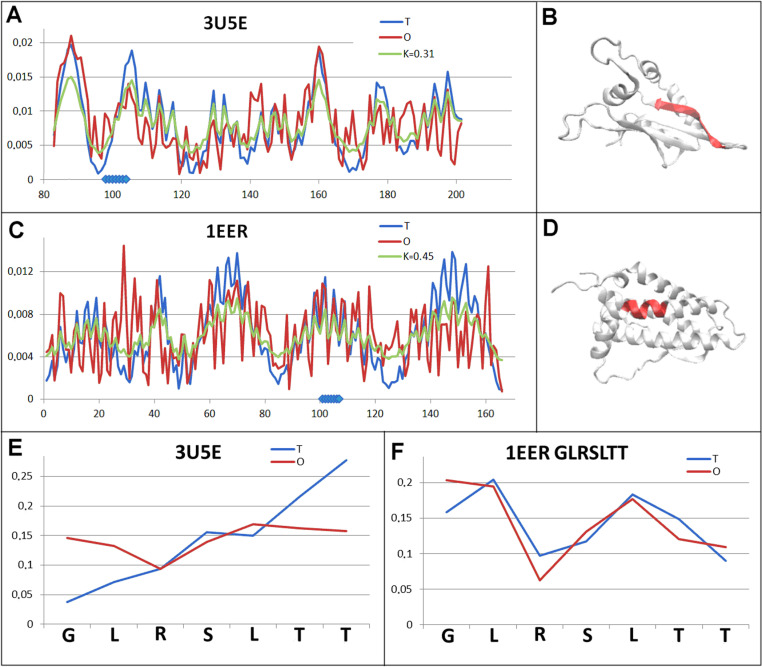
Pair characterisation (5 in Fig 3.C) - – the pair discussed is on the position 683 in ChSeq data base. A – *T, O* and *M* distribution for the *K* value (given in the legend), describing the status of ribosomal protein (3U5E). B – 3D representation of the structural unit for which the parameters have been determined with the chameleon section (red) highlighted – (3U5E). C – *T, O* and *M* distribution for the *K* value (given in the legend), describing the status of cytokine (1EER). D – 3D representation of the structural unit for which the parameters have been determined with the chameleon section (red) highlighted – 1EER. E – *T* and *O* distribution for the chameleon section in ribosomal protein (3U5E). F – *T* and *O* distributions for the chameleon section in cytokine (1EER). The blue dots on X-axis in A and C – chameleon fragment.

Another example is a set of two proteins: a chain that is part of ribosome (3U5E) and cytokine (1EER). The first protein shows an ordering expressed by *RD* <  0.5 with a low *K* value (Table 2., Fig 8.A), while the other protein of the pair is described with an *RD* value >  0.5, although this threshold is exceeded to a relatively small extent (Table 2, Fig 8.C). The status of the chameleon sections, however, is significantly different with the *RD* value for ribosomal protein being very high, while in cytokine, this status is expressed by a very low *RD* value (Table 2.) (Fig 8.E and 8.F).

In the case of ribosomal protein, local mismatches are apparent, mainly in the form of increased surface hydrophobicity exposure (Fig 8.A – the *O* distribution line above the T distribution line). Sections with hydrophobicity ordering consistent with the expectation of a micelle-like arrangement are also present. The status of the chameleon section in ribosomal protein is very specific, where, in addition to the three central positions showing a comparable status (*O* versus *T*), it is the N- and C-terminal positions of this chameleon section that show extremely opposite levels (Fig 8.E).

A different status is observed in cytokine, where the chameleon section with the helical form clearly shows an amphipathic arrangement where high levels (presumably directed towards the inside of the protein body) overlap with the O status. Similarly, the expected increased level of hydrophobicity is realised by the corresponding amino acid arrangement in this section.

**Fig 9:**
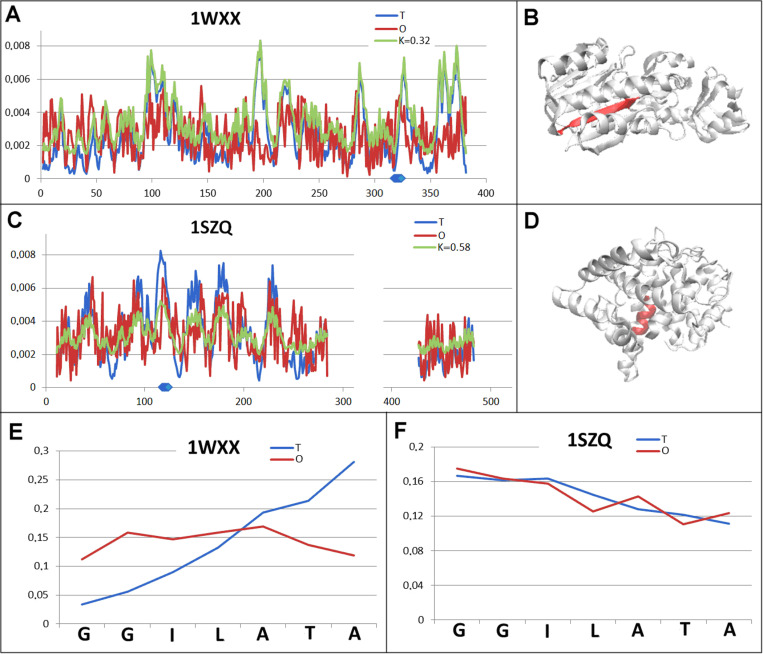
Pair characterisation (6 in Fig 3.C) - – the pair discussed is on the position 408 in ChSeq data base. A – *T, O* and *M* distribution for the *K* value (given in the legend), describing the status of transferase (1WXX). B – 3D representation of the structural unit for which the parameters have been determined with the chameleon section (red) highlighted – (1WXX). C – *T, O* and *M* distribution for the *K* value (given in the legend), describing the status of lyase (1SZQ). D – 3D representation of the structural unit for which the parameters have been determined with the chameleon section (red) highlighted – 1SZQ. E – *T* and *O* distribution for the chameleon section in transferase (1WXX). F – *T* and *O* distributions for the chameleon section in lyase (1SZQ). The blue dots on X-axis in A and C – chameleon fragment.

A group showing variation in the status of chameleon sections includes the pair: transferase (1WXX) and lyase (1SZQ). In this arrangement, transferase as a structural unit shows a rather significant micelle-like ordering, while the status of lyase clearly exceeds the discrimination threshold (*RD* >  0.5) (Table 2, Fig 9.A and 9.C).

This significant variation in the status of structural units appears to be distinct from the variation in the status of chameleon sections (Fig 9.E and 9.F). In an ordering consistent with a micelle-like arrangement in transferase, the chameleon section represents an extreme mismatch of hydrophobicity levels in transferase. The significantly incompatible arrangement towards the micelle-like arrangement in lyase contains a chameleon section in its structure perfectly reproducing the expected hydrophobicity levels (Fig 9.F).

The pair of proteins discussed here are enzymes. Their functional variation and thus their specific structure (as well as their specific arrangement within the internal force field originating from the protein body surrounding the catalytic site) justifies the observed variations. The position of the point (Fig 3. – example 6) can be explained by the high variation in specificity within the biological activity of the compared proteins.

#### An example representing the very high similarity status of the chameleon sections representing micelle-like ordering.

**Fig 10:**
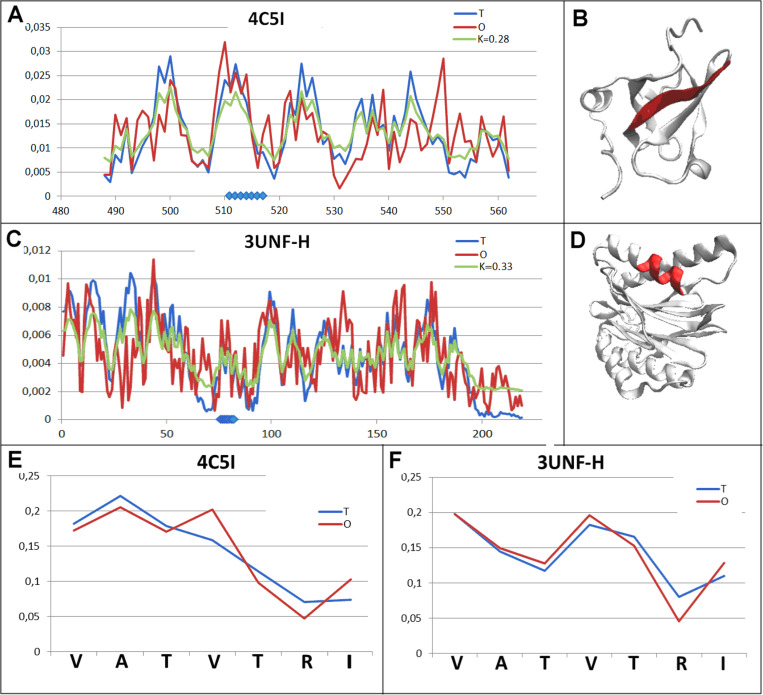
Pair characterisation (7 in Fig 3.C) - – the pair discussed is on the position 237 in ChSeq data base. A – *T, O* and *M* distribution for the K value (given in the legend), describing the status of transferase (4C5I). B – 3D representation of the structural unit for which the parameters have been determined with the chameleon section (red) highlighted – (4C5I). C – *T, O* and *M* distribution for the *K* value (given in the legend), describing the status of lyase (3UNF-H). D – 3D representation of the structural unit for which the parameters have been determined with the chameleon section (red) highlighted – 3UNF-H. E – *T* and *O* distribution for the chameleon section in transferase (4C5I). F – *T* and *O* distributions for the chameleon section in lyase (3UNF-H). The blue dots on X-axis in A and C – chameleon fragment.

The final example is a set of two chameleon proteins: Transcriptional repressor protein yy1 (4C5I) and hydrolase (3UNF-H). The uniqueness of this pair of proteins lies in representing the status of the structural units of the forms described by *RD* values close to the *RD* = 0.5 level with comparable *K* values (Table 2, Fig 10.A and 10.C). What is surprising, however, is the extremely high match of the local *O* distribution (within chameleon sections) with the *T* distribution. Comparable structural units, despite the significant difference in the length of the chains that build them, show a comparable status. In contrast, the statuses of the chameleon sections show an extremely high match between the *O* distributions and the expected *T* distributions in these structural units (Fig 10.E and 10.F). This happens when a biological function is significantly different.

This set of chameleon proteins, together with the set presented in[25] provides significant support for the hypothesis proposed regarding the subordination of structuring at the secondary structure level to structuring of the whole unit, which is expected to perform a specific biological function. The sections with a specific secondary structure act as a comparable local tool for the provision of this superior role, which is the biological function of the structured unit.

## Discussion

The topic discussed herein remains in close relation to the analysis of structure dependence on amino acid sequence. The proteins representing opposite structural forms (taking secondary structure as the criterion). It is shown that the mutation on one single position in the sequence of 56 aa causes radical change of helical structure to β-structural form [43–45]. The interpretation of the status of both proteins reveals the role of hydrophobicity organisation in protein body. The FOD model applied identifies in both proteins very similar organisation of hydrophobicity in proteins bodies [46]. The water-directed structuralisation produced micelle-like distribution in both proteins independently on secondary structure [47].

The application of the FOD model to analyse the structures of these experimentally derived proteins revealed a structuring mechanism leading to micelle-like ordering in both forms, which was achieved by means of matching secondary structures [47–51].

The examples given herein suggest the pursuit of structuring directed by the environment (polar water), achieving, independently of the dominant secondary structure (helical, Beta-structure), high micelle-like ordering with a centrally located core and polar surface. These observations provide the basis for the hypothesis proposed herein about the secondary role of the secondary structure in relation to the ordering expected in a given protein, understood as the appropriate ordering of hydrophobicity levels in the protein body.

Proteins containing chameleon sections represent an important object in the analysis of the sequence relationship to secondary structure. The current results indicate that structuring follows a scenario in which the expected biological role is achieved independently of the secondary structure of sections with an identical amino acid sequence.

The assessment of the status of the chameleon sections in the sub-base discussed here as comparable irrespective of the secondary structure seems to confirm the assumed hypothesis. This is particularly relevant given that the structural units containing the chameleon sections represent a significantly different ordering from the point of view of the type of hydrophobicity ordering. Here, the structural units represent a significantly different status (*RD* <  0.5 in one pair unit and *RD* >  0.5 in the other pair unit).

The relatively abundant representation of enzymes in the group of chameleon protein pairs discussed in the present work (especially within examples 3–6) is significant. These proteins showing high specificity (subordination of structuring to biological function) acquire a structure with a significant degree of involvement of the specific environment (abundant representation with structures described by high *RD* values and high *K* values [52]). A high (and even unpredictable) variability of environmental conditions (variability of the *K* parameter) can be assumed, hence the structural variability of the enzymes may also have entailed a high variability of the status of the chameleon sections. However, the representation of such highly variable status is very sparse (outlier points).

A special place in the analysis of structural transformation is occupied by amyloid transformation, which involves structural forms with a dominant Beta-structure [53–56].

Context-dependent structure formation expressing the postulate of non-local targeting versus broader context-dependent adoption of the corresponding secondary structure form was presented in [57].

Thus, it was shown that the sequence itself can acquire different secondary forms as a result of the dependence of protein folding on environmental factors.

The comparison of native and amyloid structural forms (both structures solved: α-synuclein, V-domain of light chain of IgG and transthyretin) suggest the significant importance of environment characteristics and its influence on this transformation – as it is revealed using FOD-M model [22,58–60].

It should be noted that the environmental conditions for this transformation show some specificity in the form of the contribution of air-water interphase favouring the formation of a system with a flat (2D) structure of each single chain in amyloid fibril [61]. The value of the correlation coefficient for 90% of pairs expressing a uniform representation of the status of the chameleon sections with a highly variable status of the structural units corroborates the hypothesis of structure selection from the point of view of the organisation of the entire structural unit rather than amino acid preference for the secondary structure. This observation is all the more important, as the status of structural units containing chameleon sections in their sequence is highly variable (pairs of chameleon proteins show *RD* >  0.5 and *RD* <  0.5). This means that in proteins (structural units) adopting a micelle-like form (the folding process directed by the polar water environment) as well as those where (according to the FOD-M model assumptions) the contribution of a non-aqueous local environment for the folding process, the status of the chameleon sections is comparable irrespective of the secondary form.

The other two sub-bases extracted from the ChSeq database: pairs of proteins with a status expressed by *RD* <  0.5 for both proteins in the pair (in preparation) and the other with a status expressed by *RD* >  0.5 [25] indicate a similar conclusion by showing high values of correlation coefficient for the expression of status in the two chameleon pair proteins compared.

The presented model was applied to many proteins representing different structure, different biological activity. The summary of these analyses deliverd in conclusion the modified form of funnel model [62]. The quantitative form of horizontal axis in funnel model is expressed by variable representing K values different energy minima. The treatment of protein folding as multiple object optimisation reveals the necessary dependence of final energy level as dependent on two functions: internal force field (non-bonding interactions between atoms present in protein - f_INT_(r_ij_)) and external force field (influence of the environment which is expressed in FOD-M model as the continnum - f_EXT_(r_ij_, K)):


$$\text{F}\left({\text{r}}_{\text{ij}}\right)\text{= F}\left({\text{f}}_{\text{INT}}\left({\text{r}}_{\text{ij}}\right){\text{, f}}_{\text{EXT}}\left({\text{r}}_{\text{ij}}\text{, K}\right)\right)$$ (7)

The future plans are focused on the simulation of proteins in conditions defined by different external force field (different forms of M function – eq. 6). It is assumed and expected to receive also structures present in amyloid fibrils as the effect of folding simulation (or structural transformation) in conditions expressed by appropriate K values defining the form of external force field [63,64].

## Conclusions

The present work demonstrates the superior role of the structure of a hydrophobicity distribution system common to the whole protein molecule (structural unit), subordinating the attainment of a structure that guarantees adequate biological activity. The adoption of an appropriate form of secondary structure is of lower importance, subject to the mechanism of the folding process under conditions that may be different from the aqueous environment (variation in environmental specificity – parameter *RD* <  0.5 in one protein of a chameleon protein pair with *RD* >  0.5 in the other protein of the pair). Both a micelle-like status and a status far from a micelle-like arrangement are achieved with adaptation of the appropriate form of the hydrophobicity distribution of the chameleon section independently of the secondary structure of this section. Different external conditions (external force field) actively involved in the folding process appear to play a dominant role in relation to the predisposition of the amino acid sequence itself. The multiple object optimisation method applied in form of Front Pareto model is expected to deliver the environment-dependent energy minimisation procedure [65]. It is also expected to receive the structures appropriate for proteins acting in their specific external conditions (membrane, water) as well as those requiring the presence of assistance proteins chaperone or chaperonin) to receibve the structural forms ensuring the biological activity. The folding of chameleon proteins is also expected follow the environment dependent mechanism of folding.

## Supporting information

S1 File The Supporting Information describes the calculation procedure to allow the recalculation of presented results.
(DOCX)
