# Supplementary material for: Chameleon sequences—Structural effects
Source: PLoS One. 2025 Apr 22;20(4):e0315901. doi: 10.1371/journal.pone.0315901 (PMC12013887; doi:10.1371/journal.pone.0315901)
Supplement: S1 File — The Supporting Information describes the calculation procedure to allow the recalculation of presented results. (DOCX) [file pone.0315901.s001.docx]

SUPPORTING INFORMATION

**CHAMELEON SEQUENCES – STRUCTURAL EFFECTS**

**Mateusz Slupina, Katarzyna Stapor, Leszek Konieczny,** **Krzysztof Gądek, Piotr Nowakowski, Irena Roterman**

1. The proteins present in ChSeq data base
2. The sub-set defined in the data base by the option: 7 amino acids sequence of chameleon fragments was selected with the option strict match.
3. There were 777 pairs of proteins on the list as described above. (access Nov 2022)

The starting point was the ChSeq data base available currently as below:

<http://prodata.swmed.edu/wenlin/pdb_survey2/index.cgi/> [[Li](https://pubmed.ncbi.nlm.nih.gov/?term=Li+W&cauthor_id=25970262) W, [Kinch](https://pubmed.ncbi.nlm.nih.gov/?term=Kinch+LN&cauthor_id=25970262) LN , [Karplus](https://pubmed.ncbi.nlm.nih.gov/?term=Karplus+PA&cauthor_id=25970262) PA, [Grishin](https://pubmed.ncbi.nlm.nih.gov/?term=Grishin+NV&cauthor_id=25970262) NV. ChSeq: A database of chameleon sequences. Protein Sci. 2015; 24(7):1075-86. doi: 10.1002/pro.2689. , prodata.swmed.edu/chseq.

The option : „table with stringent criterion” was selected.

There are 777 pairs of chameleon proteins for 7 aa chameleon fragments.

The example for the sequence LFGLAVG is shown below.


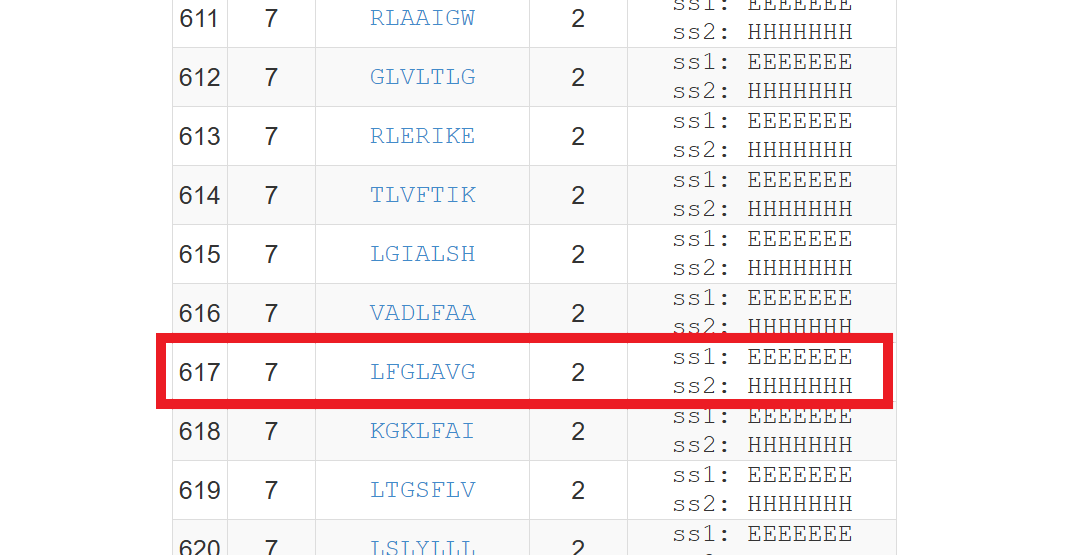


This position is represented by two proteins: 2Q80 and 3N2S what can be seen after clicking item 617 on the list as shown above. The position of chameleon fragments are given there (see below).


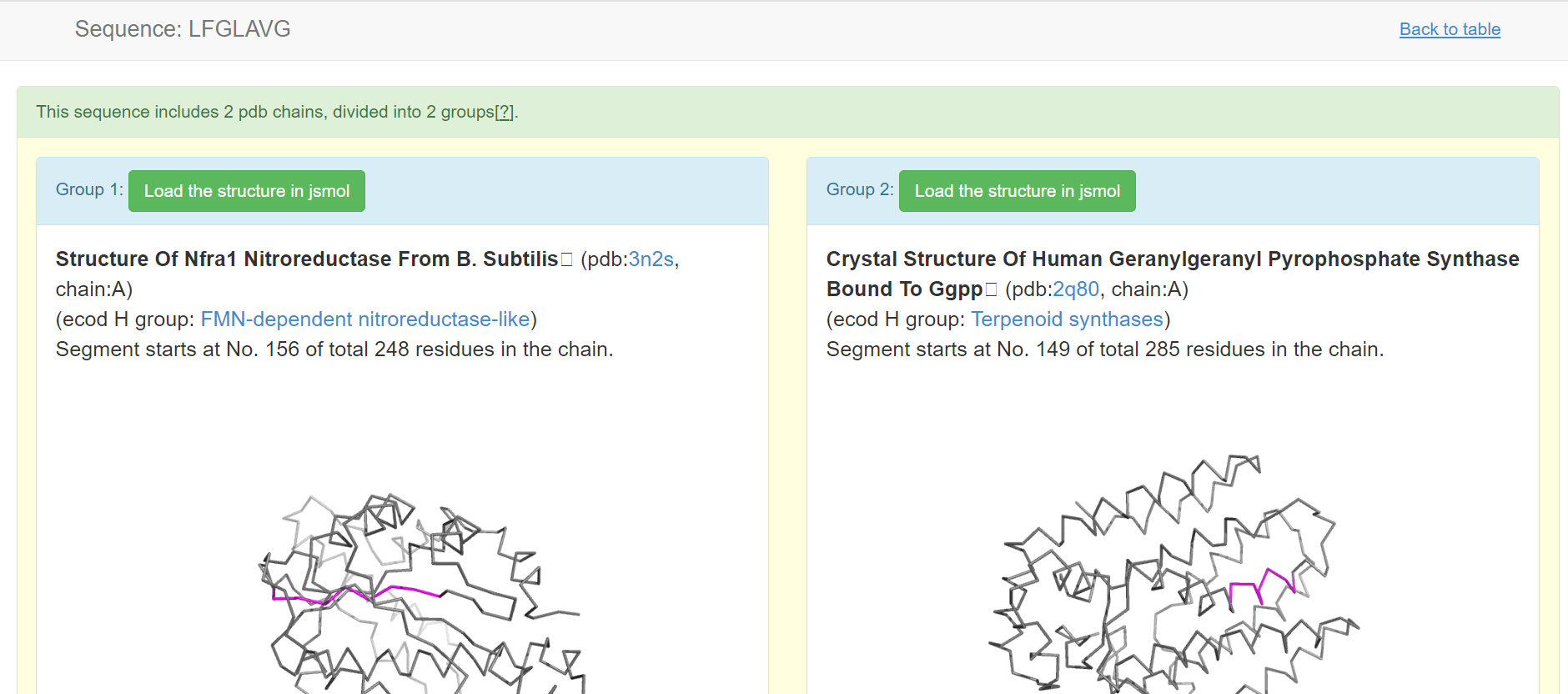


The next step is to identify the chameleon fragments in the structure of the protein. The PDBSum database uses the results of CATH analysis and provides information concerning the domain structure of each chain.

Numbers of protein pairs as they are available in ChSeq data base (according to Table - main paper).

| BETA | SEQUENCE | Position on the list  according to ChSeq | HELIX |
| --- | --- | --- | --- |
| PDB ID |  |  | PDB ID |
| 3N2S  2YGL  2J6R-B  2P8U  3U5E  1WXX  4C5I | LFGLAVG  LVLGGAL  SAAYALG  GAVALLI  GLRSLTT  GGILATA  VATVTRI | 617  243  458  583  683  408  237 | 2Q80  2PZI  4JW3-C  3LXU-X  1EER  1SZQ  3UNF-H |

The next step is to calculate the RD values for structural units (chains or domains). This calculation can be performer using the HPHOB web application, which is freely available at <https://hphob.sano.science> and open to all users.


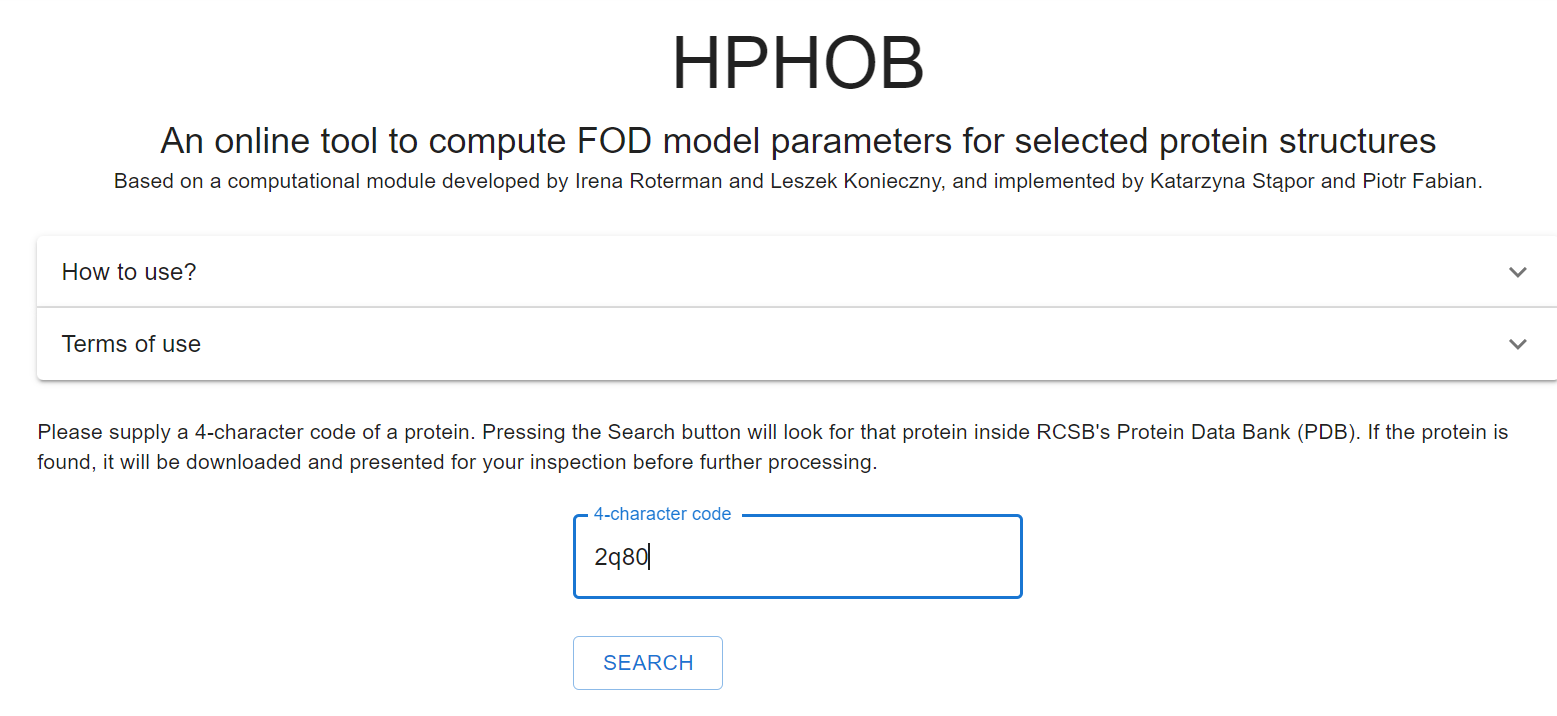


After clicking SEARCH the following screen appears.


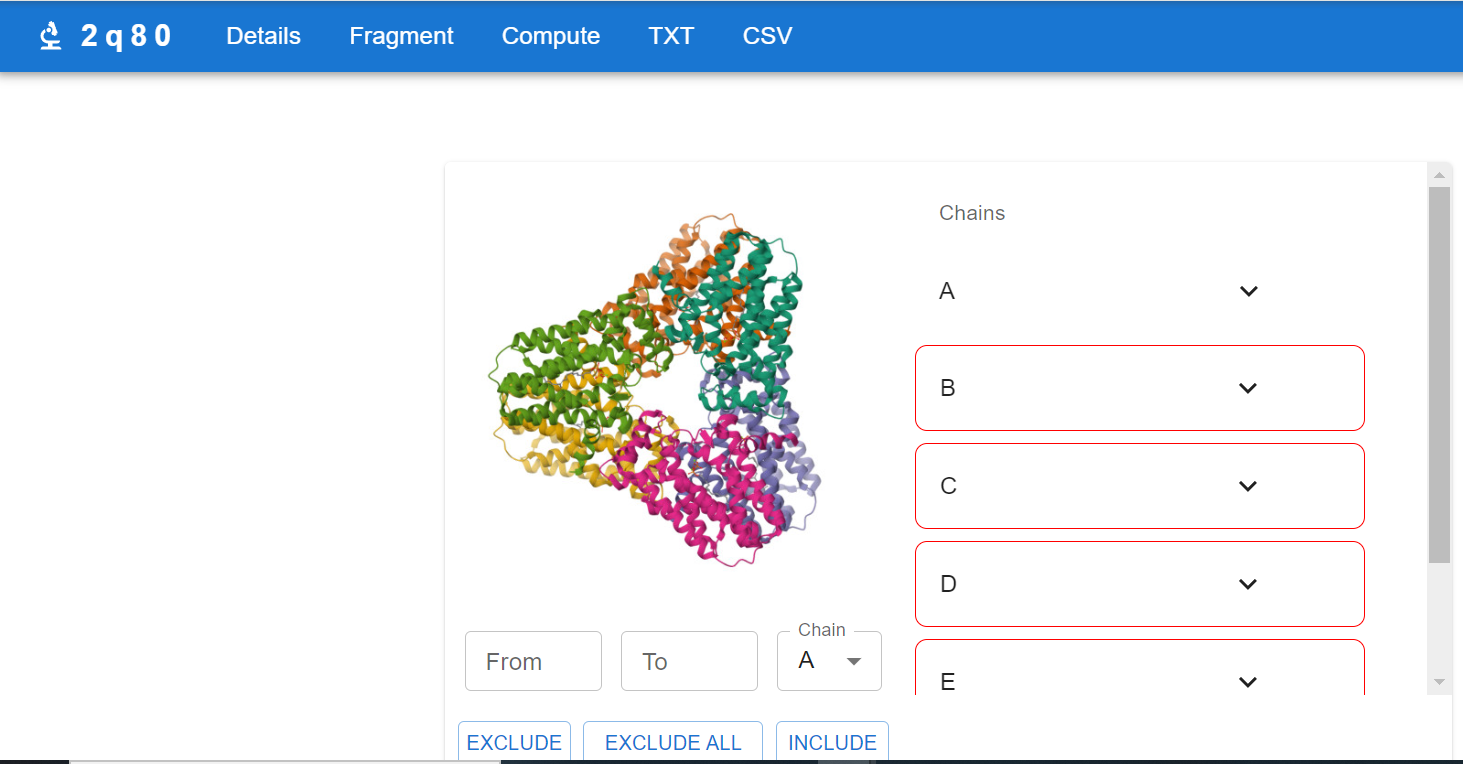


Selection of chain A is achieved by clicking EXCLUDE ALL and then INCLUDE, with chain A selected – the appropriate red frame on the right-hand side should be clicked (this action selects the given chain as the input data for calculation).

On this screen:

1. Clicking COMPUTE – the calculation will be performed for the complete molecule (complex)
2. To select a particular fragment or chain, click FRAGMENT – the previously shown screen appears

Select EXCLUDE ALL

Then INCLUDE chain A – click the red frame labeled A

Click CALCULATE

1. The result appears as shown below, listing RD and K values accordingly.


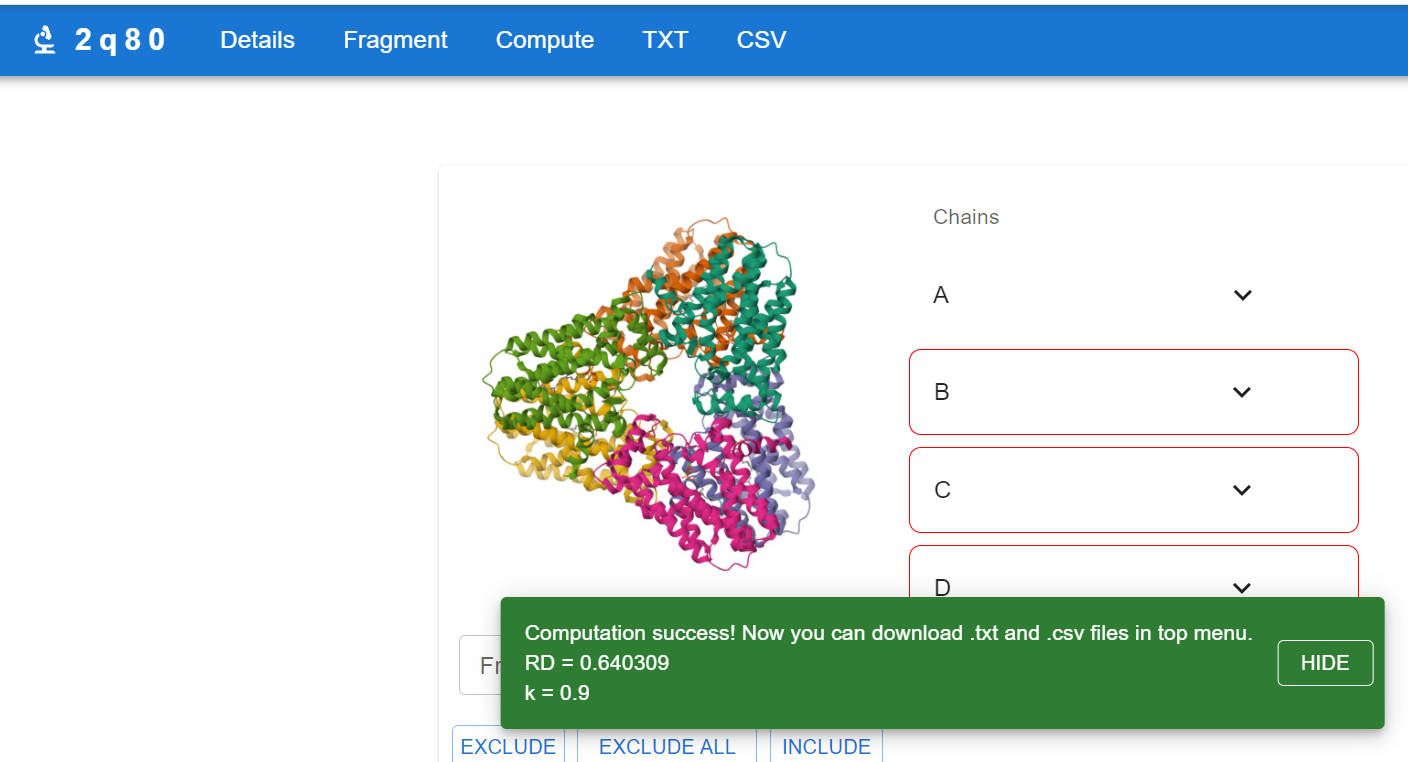


1. Results can be downloaded in the appropriate format as shown on top (blue bar).
2. The RD value may also be calculated directly, as shown below


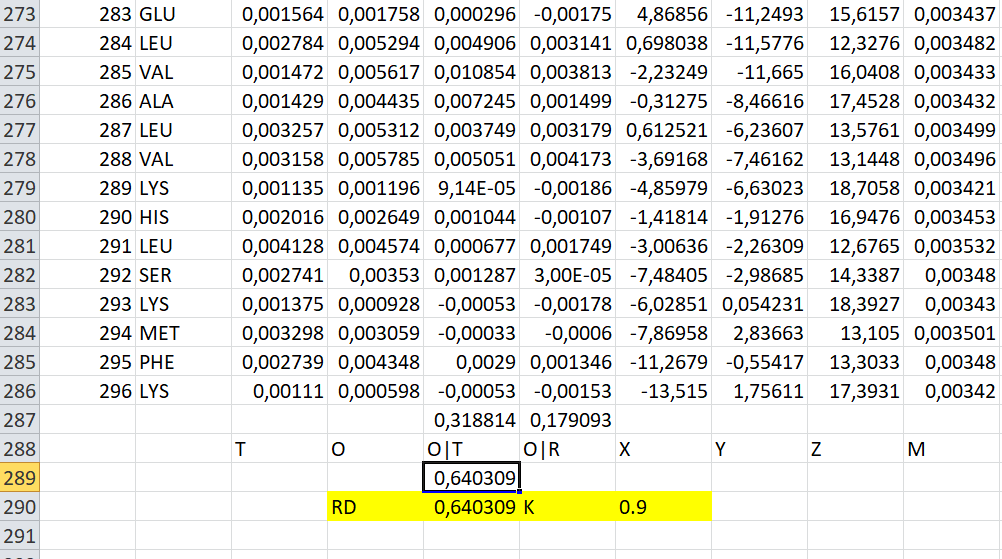


Line 287 – sum of the columns O|T and sum of O|R

Line 289 O|T / (O|T+O|R) – this is formule to calculate RD value

This is example for 2Q80 – chain A.

X, Y, Z – coordinates for effective atoms

M – profile M for K=0.9. The value of K available also in form of the output file.

Chameleon sequence LFGLAVG in 2Q80 selected (below – red)


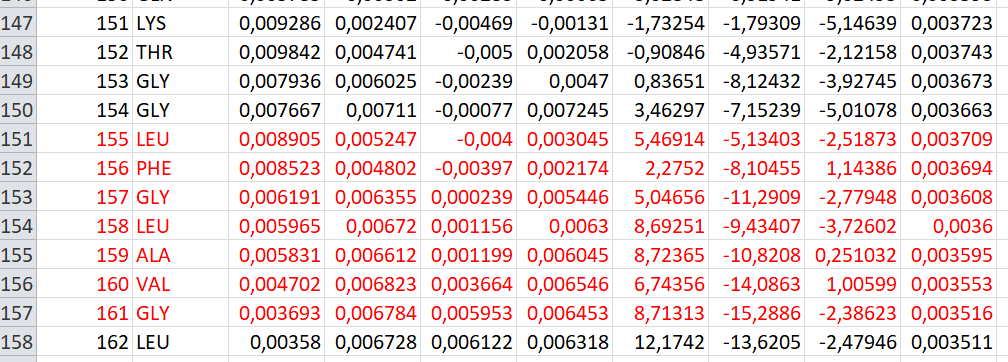


The calculation of RD for the chameleon fragment is performed manually.

The following screen shows this operation


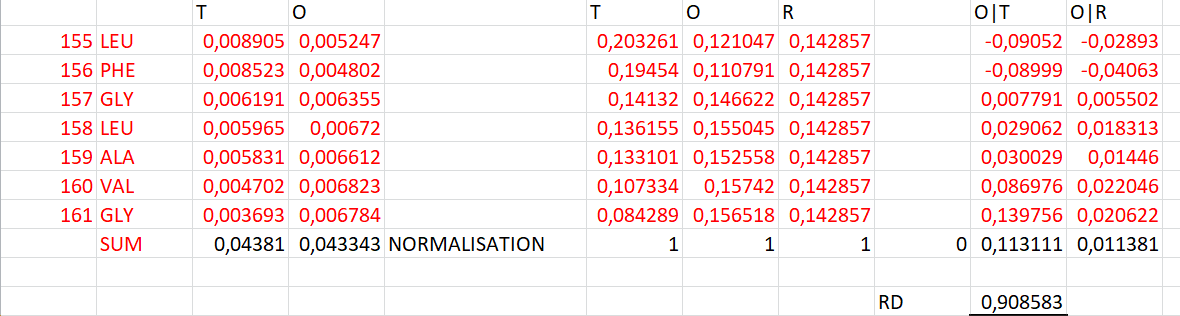


Normalization of T, O and R distributions for this fragment is depicted. Results indicate that the chameleon fragment, when treated as part of the protein molecule, does not contribute to a micelle-like structural arrangement.

The same chameleon fragment is also analyzed in 3N2S:


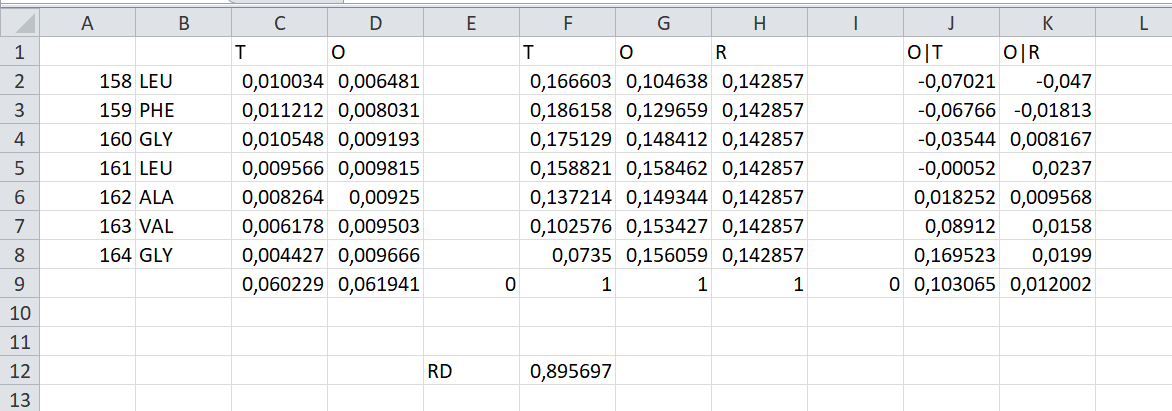


This shows that the local status of the fragment in 3N2S is similar to the one observed in 2Q80, and that the local status of both helical and Beta-structural chameleon fragments induce similar local organization of hydrophobicity in these two proteins. However, the status of the molecules as a whole is different. 3N2S is in high accordance with the micellar pattern (RD for whole chain = 0.423 with K=0.28). In contrast – 2Q80 strongly diverges from this pattern (RD for complete chain = 0.640 with K=0.91). As shown, the local status of the chameleon fragments is very similar in both cases (Fig. 4 in the paper).

General comments:

1. Mateusz Slupina, a PhD student, initially performed the calculation by himself. His calculations produced a K value accurate to within two decimal positions. The HPHOB server was set up to automate his calculations, however we decided that maintaining accuracy to within two decimal positions is not necessary. This is why the PHPOB server delivers only one decimal position. Files with original results (calculation of RD) are available in the .xlsx format for 2Q80 and 3N2S proteins.
2. If the chameleon fragment is localised in the domain, the domain shall be treated as a structural unit. The HPHOB server supports this selection. The beginning and final positions of domains shall are provided in a dialog box, as shown below.


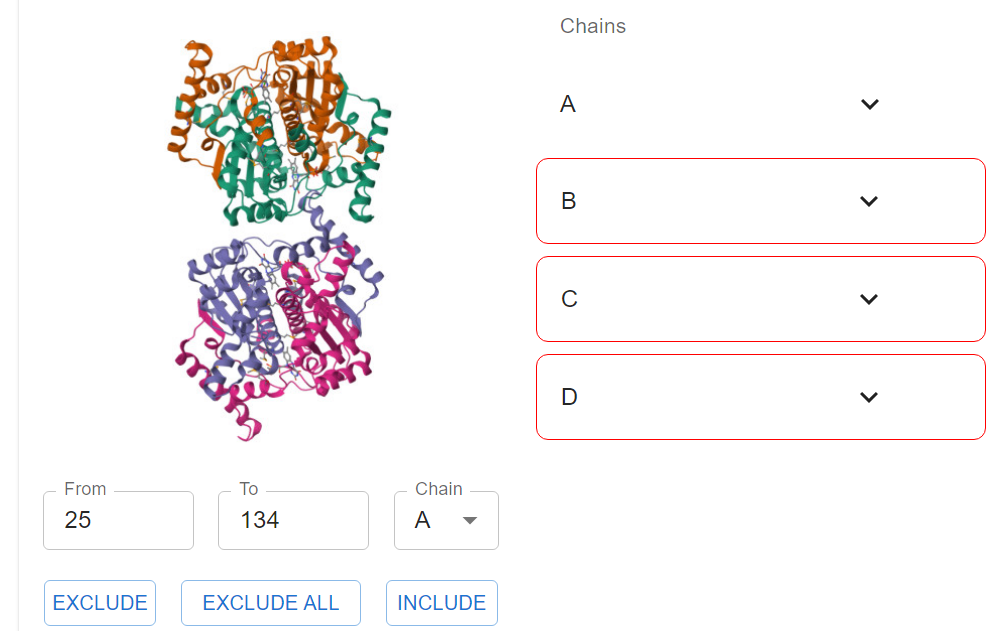


For a domain consisting of residues 25-134, the selection can be performed as above. If the domain consists of two or more fragments, the option INCLUDE can be used to assemble such a multipart domain.

1. The values received for structural units containing chameleon fragments appeared to be very different. This is why the dataset (777 pairs) was divided into three groups:

A – both proteins in the pair representing RD < 0.5

B – both structural units characterized by RD > 0.5

C – one structural unit described by RD < 0.5 and the second one by RD > 0.5. The third group is the subject of the paper proposed for publication in PLOS ONE.

Group B has also been characterized, and a paper describing this group is available: Roterman I, Slupina M, Stapor K, Konieczny L, Gądek K, Nowakowski P. [Chameleon Sequences-Structural Effects in Proteins Characterized by Hydrophobicity Disorder.](https://pubmed.ncbi.nlm.nih.gov/39310170/) ACS Omega. 2024 ; 9(37):38506-38522. doi: 10.1021/acsomega.4c03658. An in-depth analysis of the group denoted as A is in preparation.

1. Fig. 3.A (in paper) red dots – represent the status of the structural units present in the analysis. Blue dots in Fig. 3.A visualize the status of chameleon fragments in these pairs of proteins.
2. Fig. 3.B (in paper) shows the identification of outlying points. These points were eliminated in a stepwise manner until the correlation coefficient exceeded the value of 0.7.
3. Fig. 3.C (in paper) – green lines link the positions representing the status of chameleon fragments (blue dots) with the points representing the status of structural units they belong to (red dots).
